# Effect of endotracheal tube size on airway resistance and dynamic lung compliance

**DOI:** 10.1097/MD.0000000000031410

**Published:** 2022-10-28

**Authors:** Yiming Li, Yan Lu, Xinmin Tan, Zilong Zhang, Feng Feng, Ruhong Li

**Affiliations:** a Department of Anesthesiology, Affiliated Hospital of Chengde Medical University, Chengde, Hebei, China; b Capital Medical University Affiliated Beijing Shijitan Hospital, Beijing, China.

**Keywords:** airway resistance, Endotracheal tube, lung compliance, mechanical ventilation

## Abstract

There are different results on the effect of endotracheal tube (ETT) size on respiratory mechanics in patients undergoing mechanical ventilation, and there are few reports in adult laparoscopic surgery. The aim of this study was to investigate the effect of ETT size on airway resistance (RAW) and dynamic lung compliance (COMPL) in patients undergoing laparoscopic colorectal surgery. Seventy-two patients undergoing laparoscopic radical surgery for colorectal cancer under general anesthesia with endotracheal intubation were selected and divided into 3 groups (n = 24) using the random number table method Group A (ETT ID 7.0), Group B (ETT ID 7.5), and Group C (ETT ID 8.0). After mechanical ventilation, intraoperative RAW and COMPL were monitored in each of the 3 groups. In the non-pneumoperitoneal state, RAW in group ID7.0 is significantly higher than this in group ID7.5 and group ID8.0 (*P* < .05); the RAW between the 2 groups with ID7.5 and ID8.0 was not statistically significant (*P* > .05). The difference of COMPL between the 3 groups was statistically significant (*P* < .05); the COMPL of Group ID7.0 is lower than Group ID7.5, and Group ID7.5 is lower than Group ID8.0. In the pneumoperitoneal state, the RAW between ID7.0 group and ID8.0 group was statistically significant, the RAW difference between ID7.0 group and ID7.5 group, ID7.5 group and ID8.0 group not statistically significant (*P* > .05);the COMPL between the 3 groups was not statistically significant (*P* > .05). In the non-pneumoperitoneal state, the smaller the ETT internal diameter within a certain range, the higher RAW and the lower COMPL; in the pneumoperitoneal state, the RAW with the ID7.0 ETT was higher than that with the ID8.0 ETT, and the ETT size within a certain range had no effect on COMPL.

## 1. Introduction

The use of a smaller endotracheal tube (ETT) during general anesthesia intubation has the advantage of reducing the incidence of secondary intubation and post-extubation laryngeal edema.^[1]^ The use of a smaller ETT is also often forced in some patients with airway stenosis. The ETT as an artificial airway adds additional airway resistance (RAW).^[2]^ According to Poiseuille’s law: *R* = 8ηL/πr^4^, the flow resistance of gas flowing through the airway is inversely proportional to the fourth power of the airway radius. It can be speculated that with the smaller of the ETT used, increased RAW and lower dynamic lung compliance (COMPL) will occur, resulting in pneumatic injury^[3]^ and increasing the occurrence of ventilator-associated pneumonia.^[4]^ Reducing RAW and improving COMPL are ideal goals for preventing lung injury and controlling hemodynamic stability.^[5]^ However, in actual clinical practice, due to the presence of laparoscopic pneumoperitoneal and body positions, as well as individual differences, the influence of ETT size on the respiratory mechanics of patients undergoing mechanical ventilation has obtained different results. Stenqvist O et al confirmed that during routine anesthesia, there was little change in the respiratory mechanics of patients when using ETT with an inner diameter as small as 6.0 mm.^[6]^ The study of Xu Chun et al showed that the ETT with a smaller inner diameter increased the RAW of mechanical ventilation in children.^[1]^ In 1 neonatal study, RAW was significantly higher in children with catheters with an inner diameter of 2.5 mm than in catheters of 3.0 mm, 3.5 mm, and 4.0 mm.^[7]^ The study is rarely reported in laparoscopic surgery in adults. The aim of this study was to investigate the effect of different size ETTs on COMPL and RAW through laparoscopic colorectal surgery in middle-aged and elderly patients to provide a reference for clinical work. This study aims to explore the effects of ETT size on RAW and COMPL in patients with laparoscopic colorectal surgery to provide a reference for clinical work.

## 2. Materials and Methods

### 2.1. General information

The study was approved by the Ethics Committee of the Affiliated Hospital of Chengde Medical University (CYFYLL2021188), and the patients or their families signed a written informed consent. Seventy-two patients undergoing elective laparoscopic colorectal cancer surgery from October 2021 to May 2022 were selected, American Society of Anaesthesiologists II to III, height 160 to 180 cm, body mass index 18.5 to 28 kg/m^2^. Exclusion criteria: presence of severe obstructive or restrictive pulmonary function abnormalities, cardiac insufficiency (left ventricular ejection fraction <40%), history of previous related adverse diseases (such as difficult airway, bronchial asthma, etc), acute upper respiratory tract infection within 2 weeks before surgery, and preoperative hypoxia (pulse oximetry < 95%). The random number table method was used to divide into 3 groups (n = 24): group A with ID7.0 ETT (Tappa wire reinforced), group B with ID7.5 ETT (Tappa wire reinforced), and group C with ID8.0 ETT (Tappa wire reinforced). Tubes that were 0.5 mm larger or 1.0 mm smaller than the actual tube used and had no adverse effects on the patients were considered clinically acceptable.^[1]^

### 2.2. Anesthesia method

Left upper limb intravenous access was established, 0.9% saline was infused. Monitoring of electrocardiogram, heart rate, blood pressure, pulse oximetry, and PetCO2 were established. Arterial blood pressure was monitored by radial artery placement. Muscle relaxation was monitored using carescape Monitor (model:Carescape Monitor B650,GE Healthcare Finland oy) to stimulate the ulnar nerve; responses at the adductor pollicis muscle were monitored via tactile examination of train-of-four (TOF) stimuli every 5 minutes.

All 3 groups of patients underwent total intravenous anesthesia. After induction of anesthesia with pure oxygen for 3 minutes, sufentanil 0.4 μg/kg, midazolam 0.05 mg/kg, propofol 1.5 mg/kg, and cis-atracurium 0.15 mg/kg were injected intravenously. After controlling ventilation with the mask closed, the ETT is inserted under the mouth and connected to the anesthesia machine (Drager Fabius GS) and the bypass airflow monitor (model:Carescape Monitor B650,GE Healthcare Finland oy). The capsule pressure was controlled within 20 cmH2O (1 cmH2O = 0.098 kPa), and it was ensured that there was no capsule leakage when the regulator restriction valve of the anesthesia machine was at 35 cmH2O for manual ventilation. Volume ventilation mode was used, and the following settings were made according to the lung protection ventilation strategy: VT = 8 mL/kg (ideal body mass) [ideal body mass (kg) for male: height-100 (cm), ideal body mass (kg) for female: height-105 (cm)], and inspiration: expiration ratio of 1:2 without positive end-expiratory pressure. 12 breaths/min, maintain PetCO2 at 35 to 40 mm Hg, adjust respiratory rate according to PetCO2, Pplat ≤ 35 cmH2O, perform intraoperative manual lung expansion every 30 minutes. The pneumoperitoneal pressure was set at 12 mm Hg (1 mm Hg = 0.133 kPa). With intravenous propofol 4 to 12 mg·kg^-1^·h^-1^ and remifentanil 0.2 to 0.4 μg·kg^-1^·min^-1^, maintaining BIS to 40 to 60. Intermittent intravenous injection of cisatracurium 0.1 mg/kg to maintain T1 of TOF about 10%. The infusion of propofol and remifentanil was stopped at the end of the operation. The ETT was removed when the cough and swallowing reflexes were active and the secretions were aspirated.

### 2.3 . Observation indexes

The RAW and COMPL were recorded at 10 minutes after completion of endotracheal intubation (T1), 10 minutes after establishment of pneumoperitoneum (T2), 10 minutes after adjustment of trendelenburg position (T3) and 10 minutes after deflation of pneumoperitoneum (T4), and the mean of 5 consecutive values of COMPL and RAW were recorded.

### 2.4. Statistical analysis

Repeated measurement error and individual difference variance were calculated from the results obtained in the pretest, *α* = 0.05, 1-*β* = 0.90, and the minimum sample size required for each group was calculated as 15 cases, 45 cases in total, using PASS15.0 software. Increasing the sample size to overcome the drop out and withdrawal of the patients. Statistical analysis was performed using SPSS25.0 statistical software. Normally distributed measures were expressed as mean ± standard deviation $\left(\bar{x}\pm s\right)$, and 1-way ANOVA was used for comparison between multiple groups, and LSD-*t* method was used for 2-way comparison. The statistical data were expressed as examples, and the *χ*^2^ test was used. The correlation analysis between the 2 factors adopts the Spearman’s signed rank test and carries out the significance test. The difference was considered statistically significant when *P* < .05.

## 3. Result

### 3.1. Baseline information

Seventy-two patients were included in the experiment,1 patient was excluded due to difficulty in placing the ETT due to large outer diameter, 4 cases were excluded due to surgical changes, and 1 case was excluded due to missing data, finally a total of 66 patients were included in this study for research analysis. There were no statistically significant differences in the general conditions of the 3 groups (*P* > .05), Table 1.

**Table 1:** Basic information.

Group	A(n = 21)	B(n = 24)	C(n = 21)	*P* value
**Gender(Male\Female**)	13/18	16/8	14/7	.931
**Age** $(\bar{x}\pm s)$	62.29 ± 9.05	58.88 ± 11.09	61.29 ± 7.38	.457
ASAII/III	16/5	18/6	15/6	.952
BMI(kg/m^2^)	22.57 ± 2.09	22.58 ± 2.65	22.53 ± 2.24	.998

Group A (ETT ID 7.0), Group B (ETT ID 7.5), and Group C (ETT ID 8.0).

ASA = American Society of Anaesthesiologists, BMI = body mass index

### 3.2. Comparison of respiratory mechanics

The RAW of patients in group A was (8.24 ± 1.51) cmH2O/(l-s) at T1, (10.81 ± 3.60) cmH2O/(l-s) at T2, (11.05 ± 3.53) cmH2O/(l-s) at T3, and (7.71 ± 2.37) cmH2O/(l-s) at T4, and the COMPL was (64.86 ± 12.42) mL/cmH2O at T1, (33.33 ± 7.26) mL/cmH2O at T2, (30.81 ± 5.77) mL/cmH2O at T3, and (57.67 ± 11.61) mL/cmH2O at T4.

The RAW of patients in group B was (7.83 ± 1.71) cmH2O/(l-s) at T1, (9.83 ± 2.94) cmH2O/(l-s) at T2, (9.92 ± 2.72) cmH2O/(l-s) at T3, and (7.50 ± 1.87) cmH2O/(l-s) at T4, and COMPL at T1 was (73.79 ± 12.07) mL/cmH2O at T1, (37.13 ± 8.52) mL/cmH2O at T2, (34.46 ± 9.15) mL/cmH2O at T3, and (65.75 ± 13.86) mL/cmH2O at T4.

The RAW of patients in group C was (6.33 ± 0.91) cmH2O/(l-s) at T1, (8.38 ± 2.60) cmH2O/(l-s) at T2, (8.52 ± 2.34) cmH2O/(l-s) at T3, and (5.81 ± 1.08) cmH2O/(l-s) at T4, and COMPL was (81.29 ± 10.96) mL/cmH2O at T1, (37.24 ± 5.95) mL/cmH2O at T2, (35.00 ± 4.53) mL/cmH2O at T3, and (76.38 ± 11.83) mL/cmH2O at T4.

The RAW and COMPL at each moment using different size ETT are shown in Table 2.

**Table 2:** The RAW and COMPL at each moment using different size ETTs.

		A	B	C	*P* value
T1	RAW	8.24 ± 1.51	7.83 ± 1.71	6.33 ± 0.91*,**	<.001
	COMPL	64.86 ± 12.42	73.79 ± 12.07*	81.29 ± 10.96*,**	<.001
T2	RAW	10.81 ± 3.60	9.83 ± 2.94	8.38 ± 2.60*	.042
	COMPL	33.33 ± 7.26	37.13 ± 8.52	37.24 ± 5.95	.151
T3	RAW	11.05 ± 3.53	9.92 ± 2.72	8.52 ± 2.34*	.023
	COMPL	30.81 ± 5.77	34.46 ± 9.15	35.00 ± 4.53	.039
T4	RAW	7.71 ± 2.37	7.50 ± 1.87	5.81 ± 1.08*,**	.002
	COMPL	57.67 ± 11.61	65.75 ± 13.86*	76.38 ± 11.83*,**	<.001

COMPL = dynamic lung compliance = mL/cmH2O, RAW = airway resistance, cmH2O/(l-s).

* *P* < .05, comparison with Group A.

** *P* < .05, comparison with Group B.

In the non-pneumoperitoneal state, there was a statistically significant difference in RAW in ID7.0 and ID7.5, ID7.0 and ID8.0 (*P* < .05); the RAW between the 2 groups with ID7.5 and ID8.0 was not statistically significant (*P* > .05). The difference of COMPL between the 3 groups was statistically significant (*P* < .05).

**Figure 1.**
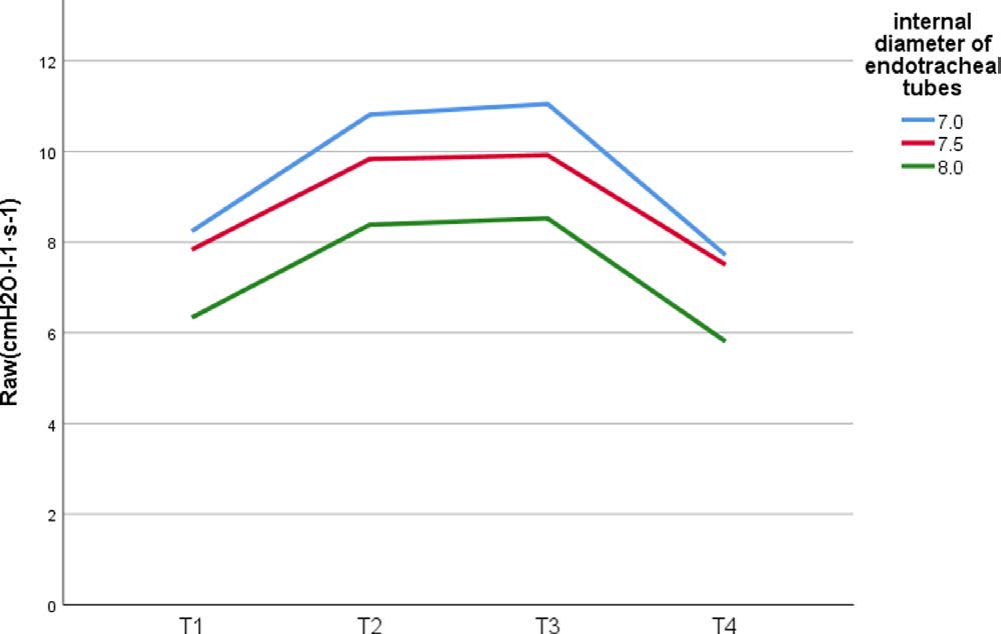
RAW at various moments with different size ETTs. ETT = endotracheal tube, RAW = airway resistance.

**Figure 2.**
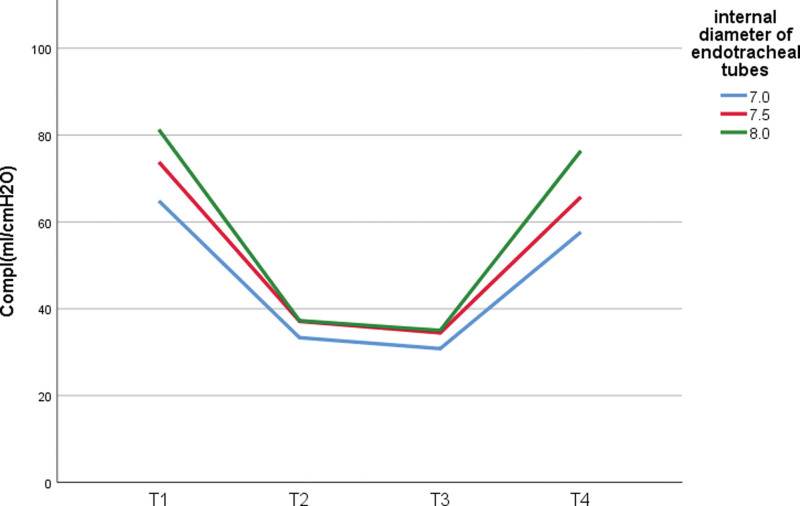
COMPL at each moment with different size ETTs. COMPL = dynamic lung compliance, ETT = endotracheal tube.

In the pneumoperitoneal state, the RAW between ID7.0 group and ID8.0 group was statistically significant, the RAW difference between ID7.0 group and ID7.5 group, ID7.5 group and ID8.0 group not statistically significant (*P* > .05); the COMPL between the 3 groups was not statistically significant (*P* > .05) (Figs. 1 and 2).

RAW and ETT size showed significant negative correlation (Spearman correlation coefficients: −0.501, *P* < .001; −0.377, *P* = .002; −0.375, *P* = .002; 0.406, *P* < .001, respectively). while COMPL showed significant positive correlation with ETT size at each moment (Spearman correlation coefficients: 0.484, *P* < .001; 0.267, P0.030; 0.291, *P* = .018; 0.538, *P* < .001, respectively) (Table 3).

**Table 3:** The correlation between ETT size and RAW and COMPL.

	T1		T2		T3		T4	
	*r*	*P*	*r*	*P*	*r*	*P*	*r*	*P*
RAW	−0.501	<.001	−0.377	.002	−0.375	.002	−0.406	<.001
COMPL	0.484	<.001	0.267	.030	0.291	.018	0.538	<.001

COMPL = dynamic lung compliance, ETT = endotracheal tube, RAW = airway resistance.

## 4. Discussion

The results obtained in clinical trials are not entirely consistent with our conclusions based on equation speculation, which may be due to individual differences, Trendelenburg position and the presence of the pneumoabdominus.

In the non-pneumoperitoneal state, the mean values of RAW decreasing with increasing internal diameter of the ETT, but the difference between the 2 groups with ID7.5 and ID8.0 ETTs was not statistically significant. The possible reason for this is that although a larger ETT provides a larger cross-sectional area, some studies have reported that a larger ETT may cause greater stimulation of the airway and endotracheal ridge, resulting in more intense bronchoconstriction, which increases RAW.^[8]^ COMPL is influenced by a variety of factors (such as the degree of myotonia, alveolar interstitial water content, degree of pulmonary fibrosis, degree of lung inflation and alveolar surface tension).^[3]^ In this study, intraoperative monitoring of myotonia with TOF and timely addition of cis-atracurium eliminated the effect of different degrees of myotonia on COMPL during mechanical ventilation. In the non-pneumoperitoneal state, the smaller ETT decreases COMPL during mechanical ventilation in patients.

In the pneumoperitoneal state, only the RAW difference between ID7.0 group and ID8.0 group was statistically significant, the RAW difference between ID7.0 group and ID7.5 group, and ID7.5 group and ID8.0 group not statistically significant, probably because the use of pneumoperitoneum during laparoscopic surgery may affect upper RAW in different ways: increased intra-abdominal pressure may redistribute blood volume, which may have a direct effect on upper airway patency by altering central venous pressure,^[9]^ or by changes in endotracheal position affecting upper RAW.^[10]^ Thus the use of pneumoperitoneum may counteract some of the direct effect of ETT ID on RAW, which is not statistically significant at a 0.5mm difference in tube size. Consistently, in an in vitro study using a lung model and an ICU ventilator also supported the use of larger size ETTs whenever possible, as larger inner diameter tubes can reduce the respiratory work of patients during autonomic breathing.^[11]^ During this period, there was no significant difference in COMPL between groups, probably because laparoscopic pneumoperitoneum leads to elevation of the patient’s diaphragm increasing the volume of pulmonary atelectasis, decreasing COMPL, and increasing airway pressure,^[12–14]^ generating endogenous positive end-expiratory pressure, which appropriately increases end-expiratory trans-pulmonary pressure, correcting to some extent the uneven distribution of gas in different regions, improving COMPL,^[3]^ with multiple factors interacting to balance the differences between the groups.

In this study, there was 1 case of postoperative hoarseness in group C. There were no abnormalities in bilateral vocal fold movements and no dislocation of the arytenoid cartilage by electronic laryngoscopy, which does not exclude that it was caused by edema of the soft tissues of the pharynx due to the larger tube. Due to the large age span observed in this study, the small number of clinical cases and the limited amount of information provided, further definitive conclusions cannot be drawn yet, and these issues will be pursued in subsequent studies. However, in obese patients, patients with bronchitis, pneumonia, COPD, and in procedures where a small ETT has to be used, we should pay more attention to changes in RAW and COMPL to reduce perioperative pulmonary complications and optimize patient outcomes. Most of the current studies on respiratory mechanics are not performed to differentiate the ETT internal diameter, and the use of correction factors for the ETT internal diameter or subgroup analysis could be considered to reduce the error and improve the credibility of the study.

## 5. Conclusion

In summary, in laparoscopic surgery, choosing a larger size ETT within the range of patient suitability is beneficial to reduce RAW and improve COMPL.

## Acknowledgments

We would like to thank all the staff in Department of Anesthesiology, Affiliated Hospital of Chengde Medical University for their contribution on our research.

## Author contributions

**Conceptualization:** Ruhong Li.

**Data curation:** Yiming Li, Feng Feng.

**Formal analysis:** Yiming Li, Feng Feng.

**Methodology:** Yiming Li, Xinmin Tan.

**Supervision:** Yan Lu, Jianling Li, Zilong Zhang.

**Writing – original draft:** Yiming Li.

**Writing – review & editing:** Yiming Li.
